# Factors influencing pre-hospital decision-making among stroke survivors in the rural area of South Africa: an exploratory study

**DOI:** 10.3389/fpubh.2026.1848220

**Published:** 2026-06-25

**Authors:** Rudzani Marry Mhlari, Bontle Kgopa, Nare Judy Masola, Tukiso Moloantoa-Sewapa

**Affiliations:** 1 School of Social Science, Psychology Division, University of Mpumalanga, Nelspruit, South Africa; 2 School of Education, Department of Educational Psychology, University of Pretoria, Pretoria, South Africa; 3 School of Health Care Science, Department of Psychology, Sefako Makgatho Health Sciences University, Pretoria, South Africa; 4 University of Limpopo, Research Administration Development, Polokwane, South Africa

**Keywords:** help-seeking behaviour, madi a magolo, medical pluralism, pre-hospital delays, rural health, South Africa, stroke, stroke survivors

## Abstract

**Introduction:**

Stroke remains a leading cause of mortality and disability globally, with a disproportionate burden in low-and middle-income countries. While early intervention is the clinical gold standard, pre-hospital delays remain a significant barrier to effective treatment in rural South African contexts. This qualitative study explored the socio-cultural, cognitive, and structural factors influencing stroke survivors’ decision-making pathways in rural South Africa.

**Methods:**

An exploratory qualitative design was employed. Ten stroke survivors were recruited from rural Limpopo Province using snowball sampling. Data were collected through semi-structured interviews and analysed using thematic analysis.

**Results:**

Three primary themes emerged as determinants of pre-hospital delay factors. Knowledge of stroke, severity of the symptoms, belief system and distance to medical health facilities.

**Discussion:**

Pre-hospital delay among rural South African stroke survivors is a complex interplay of knowledge, cultural causal attributions and systematic healthcare inequalities. Efforts to reduce pre-hospital delay must move beyond hospital-based education and enter the community’s primary networks: the home, the church, and the traditional healer’s office.

## 1 Introduction

Stroke, or cerebrovascular accident (CVA), remains a global health crisis, ranking as the second leading cause of death and the third leading cause of combined death and disability worldwide (1, 35, 36). While the clinical management of stroke has advanced significantly in high-income countries, the burden is shifting rapidly toward low and middle-income countries (LMICs), which have accounted for over 78% of stroke-related disabilities (2). Projections suggest that without a robust public health intervention, stroke-related mortality could rise to 7.8 million annually by 2030 (3). This trajectory is particularly alarming within Sub-Saharan Africa, where recent statistics indicating a 32% increase in the burden of stroke, a figure expected to rise even more sharply by 2050 (4).

In South Africa, the epidemiological landscape is undergoing a dramatic transition. Recent data from Statistics South Africa (5) indicates that cerebrovascular disease is the third leading cause of death nationally, accounting for approximately 5% of all mortality. This trend is particularly acute in rural regions, such as the Limpopo provinces, where lifestyle shifts and a lack of specialized care have led to a surge in stroke incidence (6). For those who survive, the aftermath is often devastating; nearly half of all South African stroke survivors experience permanent disability, placing an immense physical and economic strain on rural households (7).

The clinical standard for minimising stroke-related damage is rapid medical intervention. However, the efficacy of treatments like thrombolysis is strictly time-dependent, necessitating arrival at a healthcare facility within the “golden hour” of symptom onset (8). Despite this, pre-hospital delays remain the primary barrier to successful stroke outcomes across Sub-SaharanAfrica. A recent data conducted in Mozambique reported a critical delay across both prehospital and in hospital phases of stroke care (9). Similarly, in Uganda, an alarming 98% of the 110 sampled participants experienced significant pre-hospital delays (44). This trend is equally severe in South Africa, where a large-scale of 730 participants revealed that only 13% presented to a healthcare facility within 4.5 h of symptom onset. Previous research suggests that these delays are rarely the result of a single factor; rather, they are influenced by a complex web of social, cultural, and structural determinants, including symptom recognition, living arrangements, and proximity to medical facilities (9–11).

To systematically evaluate the cognitive and socio-cultural mechanisms governing pre-hospital choices, this study utilises the Health Belief Model (HBM) (45). The model posits that health-seeking behaviour is determined by an individual’s subjective perceptions of susceptibility, severity, benefits, and operational barriers, and is activated by internal or external cues to action. By framing stroke help-seeking through the use of HBM, it can be understood how symptoms, environmental factors, and cultural causal attributions can influence perceived benefits and barriers to seeking clinical care.

In many rural South African communities, health-seeking behaviour is further complicated by medical pluralism (37), the simultaneous or sequential use of Western medicine, traditional healing, and faith-based interventions. When stroke symptoms are attributed to supernatural causes or “ancestral callings,” the initial help-seeking pathway often leads away from clinical care, resulting in critical delays.

To date, few studies have qualitatively explored the lived experiences of survivors in rural areas of Limpopo, South Africa, regarding their immediate decision-making processes. This study seeks to fill that gap by exploring the factors that influence pre-hospital pathways, with the goal of informing culturally competent public health strategies that can reduce the burden of stroke in underserved populations.

## 2 Methods

### 2.1 Research approach

The study used a qualitative research approach. This approach was chosen based on the advantages that were outlined by Ospina (12): (a) qualitative research has the flexibility to follow unexpected ideas during research and to explore processes effectively; (b) it is sensitive to contextual factors; (c) it has the ability to study symbolic dimensions and social meaning; and (d) increased opportunities to develop new empirically supported ideas and theories for in-depth and longitudinal explorations of leadership phenomena and more relevance and interest for practitioners. In this regard, a qualitative approach was used to gain an in-depth understanding of the influence of delay in seeking medical help for stroke survivors.

### 2.2 Research design

This study followed the exploratory research design. The main focus of exploratory design is to explore and understand the meanings that individuals or groups ascribe to a social or human problem (13). In the case of this study, an exploratory research design helped to explore factors and reasons for delay in seeking medical help. Although research in this area exists, these factors need to be further explored to address the global crisis of this morbid condition, particularly in developing countries.

### 2.3 Sampling

Snowball sampling (also known as chain referral) was used as a sampling technique to get 10 participants. This is a commonly used sampling method in social science. It is useful when exploring sensitive and confidential topics (14). In this regard, the researcher first approached known stroke survivors to participate in the study. Such stroke survivors were then requested to identify others with a similar condition. The aim of snowball sampling is often to find and recruit hidden populations that would ordinarily not be easily accessible to researchers through other sampling strategies (15). The participants were recruited in the rural areas of Limpopo Province in South Africa. With regard to the stroke survivors who participated in the present study, the following inclusion criteria were used:

- The participants showed minimal or moderately impaired sensorimotor functioning. These are participants who can function normally after a stroke.
- The participants with reasonable mobility;
- The participants had knowledge of their clinical condition. These are participants who have been medically diagnosed and are aware of the illness.
- The participants who could communicate freely and clearly and could carry on a conversation.

The following exclusion criteria were used:

- Stroke survivors who showed severe impaired sensorimotor functioning. Survivors who lack mobility that prevents them from functioning independently.
- Individuals were unaware of the clinical condition and had not been medically diagnosed with stroke.
- Individuals with severe speech impairments and those who could not communicate freely were excluded.

### 2.4 Data collection

Qualitative data were collected using semi-structured interviews. Semi-structured interviews are a set of predetermined open-ended questions, with other questions emerging from the dialogue between interviewees (16). This allowed flexibility and more detailed information while the researcher remains focused on the research (17). Following the initial snowball referral, formal arrangements were made to meet the participants in their homes for an interview. This arrangement was to eliminate the physical burden of excessive travel and movement. Each interview was conducted strictly between the researcher and the participant to maintain confidentiality. The sessions were conducted entirely in the participant’s language of choice, which included Tshivenda, Sepedi or Xitsonga. The interviews were detailed and thorough, lasting approximately 50 to 60 min per session (Table 1).

**Table 1:** Participant’s demographic.

Participants no	Age	Gender	Occupation	Year of stroke onset
1	59	Male	Employed	2009
2	34	Female	Employed	2013
3	60	Female	Employed	2000
4	56	Female	Self-employed	2010
5	62	Male	Pensioner	2013
6	72	Female	Pensioner	2014
7	60	Male	Unemployed	2013
8	55	Female	Unemployed	2012
9	57	Male	Unemployed	2014
10	65	Female	Pensioner	2012

### 2.5 Data analysis

The data was analysed using thematic analysis. Thematic analysis provides a systematic element to data analysis and allows the researcher to associate the analysis of the frequency of a theme with the whole content (18). It allows for understanding the potential of any issue more widely (19). Thematic analysis moves beyond counting explicit words or phrases and focuses on identifying and describing both implicit and explicit ideas (20). In this study, a thematic analysis was systematically conducted following the five-phase framework outlined by Braun and Clarke (21). First, the data was transcribed and repeatedly read. Second, initial codes were generated to capture participants’ exact meanings regarding their stroke experience. Third, these codes were collated into broader, meaningful categories. Fourth, the tentative themes were reviewed and cross-checked against the raw data extract to ensure an accurate representation of the participants’ voices. Fifth, the final themes: Knowledge of Stroke, severity of the symptoms, belief system and distance to medical health facilities were clearly defined. To ensure trustworthiness and analytical rigour, the coding framework and final thematic structure were reviewed and verified by all the co-authors (42).

### 2.6 Transferability

The study was conducted with ten cohorts of stroke survivors from the rural area of Limpopo Province in South Africa. The data in this study offer critical insights into the qualitative dimensions of pre-hospital decision-making that may resonate across similar rural contexts. The findings of this study can be readily applied to evaluate help-seeking behaviours in other underserved communities facing similar structural healthcare inequality and transport limitations in rural areas.

### 2.7 Ethical considerations and procedure

Before conducting the study, the researcher obtained ethical clearance from the University Research Ethics Committee (TREC/223/2017). Informed consent: Before interviews were conducted, participants were provided with a consent letter and a form that they were requested to sign to ensure that they agree to participate in the study. Confidentiality and anonymity: The participants in the present study were assured that their information would remain confidential and that it would not be shared with anyone. Participants will remain anonymous and will not be asked for their names or any other identifying information. Debriefing for participants: Given the nature of the subject matter that was under investigation, the researcher was mindful that some participants could show some adverse emotional reactions during the investigation. Consequently, such participants who showed such adverse emotional reactions were referred to psychologists and social workers at the nearest healthcare for counselling. In this regard, during the qualitative phase, six participants were referred to the available professional resources for counselling at the local clinic.

### 2.8 Reflexivity and researcher positionality

In alignment with qualitative research principles, the researcher practiced continuous reflexivity to evaluate how her professional background, social location, and cultural identity influenced the research process from recruitment through to thematic analysis. As an academic operating within the South African higher education sector with a specialised background in psychology, she possessed the essential theoretical sensitivity and clinical empathy required to conduct sensitive, 50–60 min interview with stroke survivors. Tho mitigate potential power asymmetries, the researcher conducted all interviews entirely within the familiar domestic setting of the participants’ home, thereby reinforcing confidentiality and framing each participant as the ultimate expert of their lived experience, furthermore, the researcher’s insider status as a native speaker of the local languages allowed for a deep conceptual comprehension of unique folk categories like “madi a magolo”. Throughout the analytical process, she strictly adhered to an inductive approach, ensuring that all interpretations were anchored directly in the raw transcript.

## 3 Findings

Ten (10) participants (Male = 4; Female = 6), aged between 34 and 72 years participated in this study. Data saturation was reached by the eighth interview, as no new themes or sub-themes emerged during the final two conversations. The inclusion criteria were stroke survivors who are living with stroke or have recovered from the condition. These were stroke survivors who have been living a normal life after the stroke. Again, another inclusion from the study was participants with no speech or cognitive challenges. 3 themes were identified in this study and are discussed as follows: knowledge of stroke, severity of the symptoms, belief system and distance to medical health facilities. These themes map directly to the different components of the Health Belief Model. Knowledge of stroke and the severity of the symptoms reflect the intersection of perceived susceptibility, perceived severity, and cues to action. The belief system and distance to medical health facilities directly illustrate the tension between perceived barriers and perceived benefits in an environment characterised by medical pluralism.

### 3.1 Theme 1: the knowledge of stroke

The study found that there is a lack of knowledge about stroke and its symptoms. Most of the participants were not aware of stroke until it attacked them. Their knowledge was based on what was shared with them by the community, friends, and colleagues. Therefore, the symptoms of a stroke are not well-known and are mostly associated with other illnesses like hypertension. Hypertension, high blood pressure, and stroke are commonly classified under one name within the community. These illnesses are all referred to as “madi a magolo” (loosely high blood). This term was noted during a section at a local clinic briefing of participants waiting for chronic medication. People collectively identify stroke as “*madi a magolo*.” The warning signs of the symptoms that were common in most of the participants were itchiness in their arms, numbness in their bodies, face deformation, blindness, and dizziness. This can be illustrated in the following extract:

*“I did not know about the stroke. I was never told. When I got sick, I thought it was just a test and I stayed a little bit longer because I wanted to know what the problem was”*. [Participant 8].

*“I don’t know. It affected me twice. The first time it was in 2014. I did not know what was the problem and when it started I was outside accompanying my sister and I just felt an unusual numbness in my body and I did not know what was the problem so I continued with what I was doing. The following day I went to the doctor because the problem was not going away and that is when I was told I have stroke. I came back after that and I lived normally for the whole year and after that, one day when I woke up in the morning I could walk and my right side started not working*. *My husband took me to the hospital and when I came back I couldn’t walk properly. I have been sick ever since”*. [Participant 6].

*“I don’t know, it all started in the morning when I woke up I started cleaning my yard like I normally do and as I was busy I collapsed. I couldn’t move and talk. I just lay there”.* [Participant 4].

One participant had a first encounter with stroke when it attacked his mother and older brother. The brother is currently living with stroke and the mother has since passed away because of the illness. Because of the history of stroke in the family and other chronic diseases, the participant believes that the disease is caused by a generational curse. This means that traditionally these illnesses are understood to be the same thing. As a result, people use the same methods of treatment when dealing with any of these illnesses. This is shown in the following citations:

*“No, my mother died of a stroke so I might have inherited it from my mother. Or maybe a curse from my family because even my older brother has the same problem.”* [Participant 1].

### 3.2 Theme 2: severity of the symptoms

Lack of knowledge of the symptoms causes a delay in seeking help. Another factor that led to delay in seeking medical help for many participants was the severerity of the symptoms. Many of the participants waited for the symptoms to aggravate before seeking professional help. Most participants had a symptom for 7 days and ignored them with the hope that the symptoms would disappear on their own. She only consulted a doctor when the situation worsened. This can be evidenced by the following extract:

*“No, I thought it was just one of those days and I continued taking medication for the illness that I had already but the symptoms were not going away so the following day I went to the doctor. That is where I was diagnosed with minor stroke”.* [Participant 6].

*“When I got sick I thought it was just a test and I stayed a little bit longer because I wanted to know what the problem was. When I was not getting better we then arranged for transport to go to the clinic and I was taken to the hospital. That was the first one”.* [Participant 8].

*“The whole week I was having a strange feeling on my right chin. It was like something was itching me and I did not know what it was. But I ignored and continue working… It was in 2000 and I was diagnosed with high blood pressure. When the symptoms started I thought it was one of those days and it will go away”.* [Participant 3].

The other factor that influenced a delay in seeking help among the participants was the unavailability of another person when the stroke attacked. The participants could not go to the nearest clinic or hospital by themselves and could not make phone calls. One of the participants fell while she was home alone and was only found later in the day by her son who was returning from school. Meaning that in the event of a severe stroke, the survivor does not decide on where to seek medical help.

*“Until in the afternoon when my son came back from school. All day I was just there and no one to help me because you can see the area I am in. People hardly pass this area but that day it was worse because even my neighbours could not notice that they did not see anyone”.* [Participant 4].

*“Yes, because by then I was staying alone and couldn’t drive. So my mother came from work and drive me to the hospital”.* [Participant 2].

### 3.3 Theme 3: belief system and distance to medical health facilities

The study found that where the stroke victim would go to seek help depends on the understanding and belief of the cause of the illness. In this study, most participants believed that the illness was caused by witchcraft or some form of punishment from the ancestors. The participants consulted traditional healers first due to their belief that the illness was caused by supernatural powers. They would go to traditional healers because they believe that they will get proper spiritual answers about the meaning of the disease. Others believed that the illness was from their ancestors who were seeking their attention to communicate a certain message.

*“At church at least they answered my questions. Nothing could have been done really because this was not natural”*. [Participant 4].

*“They told me this stroke was caused by witchcraft because of jealous. You see I used to work a lot of selling and I was building a house. You see I also know that they sell medication to cause stroke and all this ugly illness. Do you know that people can buy medication that causes stroke? That is what happened to me”.* [Participant 4].

Furthermore, most of the participants lived in remote rural areas far from healthcare facilities. The distance became a contributing factor in their delay in getting medical attention. Some traveled for as far as 15 min to the nearest clinic and about 30 min to the hospital. Mostly, the rural areas do not have stable public transport, and local people with cars charge the residents a lot of money to transport them to the hospital. Going to the hospital with hired transport was exorbitant for most victims. As a result, because of the accessibility, traditional healers became the immediate access as they live within villages and their services are relatively cheap. This can be supported by the following citation:

*“Even getting to the hospital is another process if you don’t have a car. My husband had to go around looking for a car to take me to the hospital”.* [Participant 10].

*“The clinic is far so this means for me to get to the clinic it will take me time so I usually rely on African medications”.* [Participant 8].

*“I don’t want to go to the clinic every time I need medication. I have to walk all the way to the clinic and is far and I don’t have anyone to transport me. Sometimes I forget to go to the clinic to get treatment, I am only reminded when they come. I can’t walk properly and for me to get to the clinic takes time. We don’t have sufficient transport here”.* [Participant 7].

*“The hospital is very far from here and every time we will travel a distance until another sister told me that I don’t need to come to the hospital I should go to the nearest clinic to get my medication. But the clinic is still far because we don’t have transport this side”* [Participant 6].

## 4 Discussion

The findings of this study reveal that pre-hospital decision-making among rural South African stroke survivors is not merely a logistical challenge, but a complex negotiation between competing knowledge systems. The delay in seeking medical care is governed by three primary pillars: The knowledge of stroke (health literacy), socio-cultural attribution (belief systems), and structural accessibility.

### 4.1 The knowledge of stroke

A primary finding was the profound lack of stroke-specific knowledge among participants. This aligns with global trends observed in rural Montana (22) and Australia (23), where geographic isolation often correlates with limited health literacy. A unique contribution of this study is the linguistic conflation of stroke with chronic hypertension. By referring to both as “madi a magolo” (high blood), participants conceptualised stroke not as an acute medical emergency, but as a persistent lifestyle condition. Because of this, symptoms such as numbness, dizziness, or facial asymmetry are filtered through the familiar framework of chronic hypertension, not a life-threatening condition. Consequently, “madi a magolo” functions as a misaligned action. Instead of the symptom triggering immediate emergency response, it prompts self-management behaviour waiting for the symptoms to settle. The failure to differentiate between a chronic risk factor and an acute event directly leads to a “wait-and-see” approach. As participants wait for symptoms to “pass,” the window for life-saving treatment closes. This suggests that socio-economic factors like education and income are not just background variables, but active inhibitors of emergency recognition (24, 25).

### 4.2 Socio-cultural attribution

The study highlights that help-seeking pathways are dictated by causal attribution. When symptoms were attributed to supernatural forces, such as ancestral displeasure or witchcraft, the logical first step was to consult a traditional healer or the church. This medical pluralism, the simultaneous or sequential use of multiple healing systems, is a well-documented phenomenon in Sub-Saharan Africa (26, 27, 39). In this study, the preference for traditional healers was driven by their cultural accessibility and their ability to provide spiritual answers that Western medicine often ignores. Because stroke is frequently perceived as a spiritual disease (28), traditional healers become the primary gatekeepers of care. This then causes significant delays, as patients often only turn to Western medicine after traditional interventions have failed to resolve severe symptoms.

A significant structural finding was the greater accessibility of traditional healers and churches than of Western clinics. Traditional healers are embedded within the village fabric, removing the barriers of transport costs and long-distance travel. Pinkoane et al. (29) estimate that 80% of populations in developing countries live in rural areas with limited access to Western-style hospitals. Consequently, the path of least resistance leads survivors to traditional or spiritual help first. Given that traditional healers serve as an immediate, culturally accessible point of contact for rural stroke survivors, public health authorities must shift away from isolating traditional practitioners and move toward systemic, collaborative integration. Similarly, it has been recognised that traditional and biomedical health systems can productively coexist and complement one another (30, 31). Traditional practitioners share deep-rooted socio-cultural beliefs and linguistic categories within the community. This can effectively lower the perceived barriers and transform the traditional space into a life-saving gateway to clinical care.

### 4.3 Structural accessibility and barriers to medical health

Immediate medical intervention is critical for mitigating the debilitating effects of a stroke. As Marler et al. (32) and (38) emphasise, the window for effective stroke management, particularly eligibility for thrombolytic therapy, is strictly time-dependent. Despite this urgency, the majority of participants in this study faced a forced delay governed by structural inequities rather than personal choice.

In rural South Africa, distance to medical facilities is not merely a geographic measurement but a socio-economic one. Participants reported that the physical distance to healthcare facilities was compounded by a lack of reliable public transport and the high cost of hiring private vehicles. This economic barrier (41) directly influenced participants to minimise early warning signs and wait for symptoms to severely aggravate before consulting healthcare facilities (40). Individuals frequently delayed action for up to a week, harbouring the hope that minor initial symptoms would resolve on their own. As Nsereke et al. (33) suggest, when the financial cost of reaching a hospital is prohibitive, help-seeking behaviour is naturally redirected toward more affordable local alternatives. In this study, the time from onset to arrival is often dictated by the survivor’s ability to secure immediate funds and transport, rather than the severity of their medical need.

To mitigate the impact of such geographic isolation, the public health framework must shift towards targeted operational redesign. This structural approach is supported by a systematic study conducted by (43) in Canada, which demonstrated that implementing localised organisational strategies, such as a community-based emergency transport system, decentralising triage protocols, and strengthening referral networks, can significantly reduce pre-hospital time delays in securing acute medical care. While the infrastructural baseline in rural Limpopo differs from high-income contexts, the core organisational principles remain highly transferable. By organising subsidised community transit networks and equipping village-level clinic staff with streamlined referral pathways, local healthcare authorities can ensure that geographic distance no longer determines stroke survival or long-term disability.

## 5 Conclusion

The prevalence of stroke is rising in South Africa, yet the pre-hospital pathways to care remain fraught with cognitive, cultural, and structural barriers. This study demonstrates that for rural survivors, the decision to seek help is not a straightforward clinical reaction but a complex process influenced by a lack of specialised health literacy (as seen in the conflation of symptoms under the term “madi a magolo”), a reliance on medical pluralism, and the harsh realities of geographic isolation.

To effectively mitigate the morbidity and mortality associated with stroke in rural communities, interventions must be decentralised. Efforts to reduce pre-hospital delay must move beyond hospital-based education and enter the community’s primary networks: the home, the church, and the traditional healer’s office. Ultimately, improving stroke outcomes in rural South Africa requires a healthcare system that recognises and respects its patients’ cultural context while simultaneously addressing the structural inequities that hinder emergency access.

### 5.1 Limitations

- The population was dominated by those people who reside in the rural areas. Stroke survivors from urban and peri-urban locations were not represented. Stroke survivors face different challenges based on where they are situated. Therefore, the views of those in urban areas may differ. The translation of the interview guide into all the different languages that were used by the participants might have tampered with the original meaning of terms. However, the translations were done by professionals who understood the culture and language terminologies, which has ensured the accuracy of the interview.
- The study’s inclusion criteria included stroke survivors with minimal to moderate impairments. Consequently, the lived experiences of those individuals who suffered a catastrophic stroke are not presented.
- Though the ethical clearance was obtained in 2017, recent literature suggests that the challenges of infrastructure, geographical isolation, culture and economic barriers remain pervasive in rural South Africa (34). Therefore, this study provides a baseline understanding of factors influencing pre-hospital delays in underserved communities.

### 5.2 Recommendations

Based on the findings of the study, the following recommendations are made:

- *Culturally attuned public health campaigns*: Stroke awareness programs should use local terminology and address the specific belief systems identified in this study. Education should explicitly clarify the difference between chronic hypertension and the acute emergency of a stroke.
- *Training for traditional and spiritual healers*: Given their role as first-responders in rural “medical pluralism,” traditional and spiritual healers should be trained in the FAST (Face, Arm, Speech, Time) recognition tool to facilitate immediate hospital referrals. Health authorities must establish formal, culturally sensitive collaborative programs with registered traditional health practitioners.
- Community-based rehabilitation and support: Stroke services should extend to caregivers in rural areas, providing them with the emotional and logistical tools necessary to manage the long-term burden of the disease.
- *Structural infrastructure development*: Policymakers must prioritise the improvement of emergency transport services (ambulances) and road infrastructure in remote areas to ensure that “distance” ceases to be a determining factor in survival.

## Data Availability

The raw data supporting the conclusions of this article will be made available by the authors, without undue reservation.
